# Heat Shock Enhances the Expression of the Human T Cell Leukemia Virus Type-I (HTLV-I) Trans-Activator (Tax) Antigen in Human HTLV-I Infected Primary and Cultured T Cells

**DOI:** 10.3390/v8070191

**Published:** 2016-07-09

**Authors:** Marie Kunihiro, Hideki Fujii, Takuya Miyagi, Yoshiaki Takahashi, Reiko Tanaka, Takuya Fukushima, Aftab A. Ansari, Yuetsu Tanaka

**Affiliations:** 1 Department of Immunology, Graduate School of Medicine, University of the Ryukyus, Okinawa, 903-0215, Japan; m-kunihiro@emro.co.jp (M.K.); hfujii@med.u-ryukyu.ac.jp (H.F.); miya_skywalker2008@yahoo.co.jp (T.M.); ytakah3@med.u-ryukyu.ac.jp (Y.T.); reiko_tanaka@s5.dion.ne.jp (R.T.); 2 EM Research Organization Inc., Okinawa, 901-2311, Japan; 3 Laboratory of Hematoimmunology, School of Health Sciences, Faculty of Medicine, University of the Ryukyus, Okinawa, 903-0215, Japan; fukutaku@med.u-ryukyu.ac.jp; 4 Department of Pathology, Emory University School of Medicine, Atlanta, GA, 30322, USA; pathaaa@emory.edu

**Keywords:** HTLV-I, Tax, heat shock, adult T cell leukemia (ATL)

## Abstract

The environmental factors that lead to the reactivation of human T cell leukemia virus type-1 (HTLV-I) in latently infected T cells in vivo remain unknown. It has been previously shown that heat shock (HS) is a potent inducer of HTLV-I viral protein expression in long-term cultured cell lines. However, the precise HTLV-I protein(s) and mechanisms by which HS induces its effect remain ill-defined. We initiated these studies by first monitoring the levels of the trans-activator (Tax) protein induced by exposure of the HTLV-I infected cell line to HS. HS treatment at 43 °C for 30 min for 24 h led to marked increases in the level of Tax antigen expression in all HTLV-I-infected T cell lines tested including a number of HTLV-I-naturally infected T cell lines. HS also increased the expression of functional HTLV-I envelope gp46 antigen, as shown by increased syncytium formation activity. Interestingly, the enhancing effect of HS was partially inhibited by the addition of the heat shock protein 70 (HSP70)-inhibitor pifithlin-μ (PFT). In contrast, the HSP 70-inducer zerumbone (ZER) enhanced Tax expression in the absence of HS. These data suggest that HSP 70 is at least partially involved in HS-mediated stimulation of Tax expression. As expected, HS resulted in enhanced expression of the Tax-inducible host antigens, such as CD83 and OX40. Finally, we confirmed that HS enhanced the levels of Tax and gp46 antigen expression in primary human CD4^+^ T cells isolated from HTLV-I-infected humanized NOD/SCID/γc null (NOG) mice and HTLV-I carriers. In summary, the data presented herein indicate that HS is one of the environmental factors involved in the reactivation of HTLV-I in vivo via enhanced Tax expression, which may favor HTLV-I expansion in vivo.

## 1. Introduction

The Human T cell leukemia virus type-I (HTLV-I) is the first human retrovirus that is etiologically associated with adult T cell leukemia (ATL) and HTLV-I associated myelopathy/tropical spastic paraparesis (HAM/TSP) [1,2,3]. HTLV-I is prevalent worldwide with foci of high prevalence in southwest Japan, the Caribbean islands, South America and parts of Central Africa, most of which are located in subtropical and tropical regions [4]. HTLV-I is transmitted through contact with bodily fluids containing infected cells most often either vertically from mother to child via breastfeeding or horizontally in adults [5]. The total rate of developing ATL or HAM/TSP is roughly estimated to be 5% among HTLV-I carriers [6]. Although the molecular basis for the development of these HTLV-I-related disorders is still unclear, high levels of HTLV-I proviral load (PVL), as shown by the number of proviral DNA copies per 100 cells, is suggested to be one of the risk factors for the diseases [7].

HTLV-I is dormant in vivo at least in peripheral blood or lymph nodes, so that freshly isolated lymphoid cells from HTLV-I infected individuals do not express detectable levels of mRNA or proteins of HTLV-I [8]. However, the continued presence of strong CD8^+^ cytotoxic T lymphocyte (CTL) responses and readily detectable levels of antibodies specific for HTLV-I antigens in not only ATL and HAM/TSP patients but also asymptomatic HTLV-I carriers clearly indicate that repeated production of HTLV-I must occur in vivo. In accordance, once fresh peripheral blood mononuclear cells (PBMCs) of HTLV-I infected individuals are cultured in vitro for a short time, they begin to produce HTLV-I antigens. Little is known, however, about the mechanism(s) for HTLV-I activation in vivo. So far, several lines of in vitro studies have shown that HTLV-I is activated upon host cell activation by a variety of stimuli. These include lactoferrin [9], prostaglandin E2 (PGE2) [10], T cell activation agents such as anti-CD3 plus anti-CD28, anti-CD2 antibodies, mitogens including phytohaemagglutinin (PHA) and phorbol myristate acetate (PMA) [11,12,13], and agents that induce cellular stress-including HS and oxidation [14,15].

It is well established that heat shock proteins (HSPs) play roles not only in housekeeping functions by serving as molecular chaperones for cell survival but also in supporting gene expression of various DNA and RNA viruses [16]. According to the geographic bias of HTLV-I prevalence towards regions in subtropical and tropical areas and the finding that HS up-regulates HTLV-I synthesis in infected T cell lines in vitro [14,15], we hypothesized that prolonged exposure to strong sunlight may be one of the environmental factors for HTLV-I reactivation in vivo. This view is supported by the fact that infrared radiation that accounts for approximately 40% of the solar radiation energy that reaches the ground leads to the generation of heat and increases skin temperature at a level that induces HS [17]. Because HTLV-I production is dependent on its enhancer onco-protein, trans-activator (Tax), it may be possible that a thermal shock stimulates Tax expression at first followed by trans-activation of HTLV-I structural proteins and activation of Tax-inducible cellular proteins including OX40 and CD83 [18]. In accordance, it has been shown that HTLV-I Tax protein does associate with a number of HS proteins, such as HSP90 and HSP70 [19,20].

Using our library of anti-HTLV-I monoclonal antibodies (mAbs), and ATL patients’ and HTLV-I carriers’ blood samples, we determined the effect of HS in more detail by studying the expression of HTLV-I antigens in various HTLV-I-infected T cell lines and primary human CD4^+^ T cells. We report herein that HS significantly enhanced the expression of Tax followed by enhanced expression of gp46 along with Tax-inducible host proteins. These results reported herein suggest a role for environmental heat stress on HTLV-I reactivation in vivo.

## 2. Materials and Methods

### 2.1. Reagents

The medium used throughout the studies consisted of RPMI 1640 medium (Sigma-Aldrich Inc., St. Louis, MO, USA), supplemented with 10% fetal calf serum, 100 U/mL penicillin and 100 μg/mL streptomycin (hereinafter called RPMI medium). The IL-2-dependent T cell lines utilized herein were maintained in vitro in RPMI medium containing 20 U/mL of recombinant human IL-2. Our mouse and rat monoclonal antibodies (mAbs) utilized in this study included mouse IgG3 anti-HTLV-I Tax (clone Lt-4) [21], rat IgG2a anti-HTLV-I gp46 (clone LAT-27) [22], and mouse anti-OX40 (clone B-7B5) [23]. These in-house mAbs were purified from the ascites fluids of CB.17-SCID mice by ammonium sulfate precipitation followed by gel filtration using Superdex G-200 (GE Healthcare, Tokyo, Japan). Aliquots of these mAbs were labeled with fluorescein isothiocyanate (FITC), HyLite Fluor™ 488, HyLite Fluor™ 647 or HRP (Dojindo, Kumamoto, Japan) according to the manufacturer’s instructions. Anti-human CD4 and CD83 mAb were purchased from Beckman Coulter, Inc. (Brea, CA, USA) and BioLegend (SanDiego, CA, USA), respectively. Anti- HSP70 antibody (clone: C92F3A-5) was purchased from StressMarq Biosciences Inc (Victoria, BC, Canada). Zerumbone (ZER) [24] and pifithlin-μ (PFT) [25] were purchased from Focus Biomolecules LLC (Plymouth Meeting, PA, USA) and Sigma-Aldrich. Inc, respectively. ZER and PFT were dissolved in DMSO and stored at −20 °C until used. Mitomycin-C (MMC) was purchased from Kyowa Kirin (Tokyo, Japan) and used for the inactivation of live cells by incubation of cells at 50 μg/mL in RPMI medium at 37 °C for 30 min. Arsenite (As_2_O_3_) and N-acetylcysteine (NAC) were purchased from Sigma-Aldrich, Inc. (St. Louis, MO, USA). Cell proliferation was evaluated using the Cell Counting Kit-8 (CCK-8) (Dojindo Laboratories, Kumamoto, Japan).

### 2.2. Cell Culture and Heat Shock (HS)

The IL-2-dependent T cell lines utilized herein included an ATL patient-derived CD4^+^ ILT-H2 and the HAM/TSP patient-derived CD8^+^ ILT-M1 [26]. Other CD4^+^ T cell lines utilized were established in our laboratory from ATL patients and included ATL-026i, ATL-056i, ATL-083i, and the cell line YT/cM1 that was established from CD4^+^ T cells isolated from a normal donor. The HTLV-I-unrelated T cell line that was used for the syncytium formation assay was the Jurkat cell line (ATCC, Rockville, MD, USA) [26]. An ATL-derived IL-2-independent and HTLV-I-producing B cell line, ATL-040 established in our laboratory was used for in vivo infection of NOD/SCID/γc null mice (NOG mice) with HTLV-I. The JPX-9 cell line in which HTLV-I Tax antigen can be induced by cultivation in the presence of 10 μM cadmium (Cd) was used to determine the effect of HS on the expressions of Tax-inducible CD83 and OX40 [18].

For HS treatment, cells in growth media in a volume of 1 mL in 15 mL plastic conical tubes (BD Biosciences, San Diego, CA, USA) were heated in a water bath at various temperatures for various times as indicated in the text. After heating, these cells were dispensed into 24-well culture plates (BD Biosciences, San Diego, CA, USA) and cultured at 37 °C for 12~72 h. The protocol for the use of human PBMCs and animals were approved by the Human Institutional Review Board and the Institutional Animal Care and Use Committee on clinical and animal research of the University of the Ryukyus, prior to initiation of the present study.

### 2.3. Flow Cytometry (FCM) Analysis

The polychromatic phenotypic analysis of cells was performed as described previously [18,26]. Briefly, live cells were first Fc receptor-blocked with 1 mg/mL normal human IgG in FACS buffer (phosphate-buffered saline (PBS) containing 0.2% bovine serum albumin (BSA) and 0.1% sodium azide) for 15 min on ice. For cell surface staining, aliquots of these cells in a 96-well U-bottom plate were incubated with a panel of mAbs for 30 min on ice. For intra-cellular Tax staining, cells were fixed in 4% paraformaldehyde (PFA) for 5 min and washed in FACS buffer containing 1% BSA and 0.5% saponin, and then aliquots of these cells were stained with HyLite Fluor™ (AnaSpec Inc., Fremont, CA, USA) 647-labeled anti-Tax mAb (Lt-4) for 30 min on ice. Negative control cells were stained with fluorochrome-labeled Lt-4 in the presence of 100 μg/mL of unlabeled Lt-4 as shown previously [18]. Cells were washed and re-suspended in 1% PFA-FACS buffer, and analyzed using FACS Calibur (BD Biosciences, San Diego, CA, USA). Data was collected on a minimum of 100,000 events and analyzed using either the FlowJo (TreeStar, Inc., Ashland, OR, USA) or CellQuest software (BD Biosciences, Version 6.0).

### 2.4. Syncytium Formation Assay

The syncytium formation assay was performed as reported previously [26]. Briefly, YT/cM1 cells with or without HS treatment followed by in vitro culture for 24 h were co-cultured with an equal number of Jurkat cells in 96-well U-bottom plate at 37 °C. After 15 h, the cell cultures were transferred into a 96-well flat-bottomed well plate and syncytia were counted in 5 randomly selected fields of each well at a magnification of 100× and the mean values calculated.

### 2.5. Enzyme-Linked Immunosorbent Assay (ELISA)

Tax antigen concentration in whole cell lysates were determined using our in-house formulated and standardized enzyme-linked immunosorbent assay (ELISA) kit using a pair of anti-Tax mAbs that included the capture TAXY-7 mAb [27] and the detector HRP-labeled WATM-1 mAb [28]. Recombinant Tax protein (Proteintech, Rosemont, IL, USA) was used as a standard. The sensitivity of the assay was determined to be 0.5 ng/mL. Cell lysates were prepared by lysis of cells with a low-salt extraction buffer on ice for 30 min [29]. Protein concentration of each cell lysate sample was determined using Quick Start protein assay kit (Bio-Rad Laboratories, Hercules, CA, USA).

### 2.6. Primary human CD4^+^ T Cells Infected with Human T Cell Leukemia Virus Type-I (HTLV-I)

The protocol for infection of humanized mice with HTLV-I has been described previously [30]. Briefly, purified PBMCs (4 × 10^7^ cells/mouse) from HTLV-I-negative donors were transplanted into the peritoneal cavity of NOG mice together with MMC-treated HTLV-I producing ATL-040 cell line (1 × 10^7^ cells/mouse). Two weeks later, cells in the peritoneal lavage were collected, either untreated or heat-treated, and cultured for 1~2 days. The expression of Tax antigen in CD4^+^ human T cells was analyzed using flow cytometry (FCM) on day 1 and 2. In addition, whole heparinized peripheral blood of HTLV-I carriers was diluted 1:4 in RPMI medium, aliquoted, and an aliquot heat treated and another sham treated, and both cultured. The cells in the cultures were stained with anti-CD4, anti-Tax and anti-gp46 mAbs, and analyzed using FCM after hypotonic lysis of the red blood cells.

### 2.7. Statistical Analysis

Data were tested for statistical significance by either paired or unpaired Student’s *t* test using Prism software (GraphPad Software, Version 4.03). Data from more than three-armed experiments were analyzed by one-way analysis of variance (ANOVA) with post hoc Holm test and Tukey test.

## 3. Results

### 3.1. HS Up-Regulates the Expression of the HTLV-I Trans-Activator (Tax) Antigen

**Figure 1:**
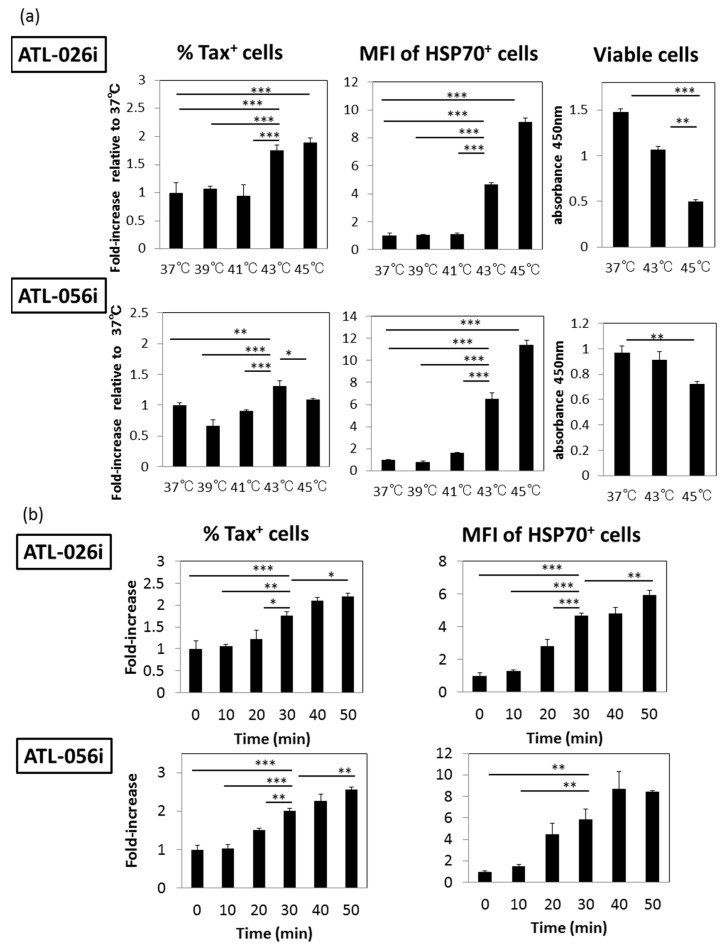
Effects of heat shock (HS) exposure on human T cell leukemia virus type-I (HTLV-I)-infected cell lines derived from acute adult T cell leukemia (ATL) patients: (**a**) Aliquots of ATL-026i and ATL-056i cells were incubated at various temperatures for 30 min and cultured for 24 h. The cells were then analyzed for the frequencies of trans-activator (Tax)^+^ cells (left bar graphs) by flow cytometry (FCM) and the relative density (Mean Fluorescent Intensity, MFI) of heat shock protein 70 (HSP70) expression (middle bar graphs) and for cell viability using the CCK-8 cell counting kit (right bar graphs). (**b**) The kinetics of the up-regulation of Tax and HSP70 expression by the same two cell lines following exposure to 43 °C for various times is shown. The values denote the means ± SD. * *p* < 0.05, ** *p* < 0.01, *** *p* < 0.001.

At first, in order to determine whether HS affects the expression of Tax antigen in HTLV-I-infected T cells, we examined two IL-2-dependent CD4^+^ T cell lines generated from acute ATL patients, ATL-026i and ATL-056i. Aliquots of these cell lines were heated at various temperatures, 37, 39, 41, 43 and 45 °C for 30 min and cultured for 24 h. The intra-cellular expression of Tax and HSP70 antigens was analyzed by FCM. Figure 1a shows that while the frequencies of Tax-expressing cells increased by HS at 43 and 45 °C in the ATL-026i cell line, the ATL-056i cell line had a broader range from 39~45 °C for Tax expression. The enhanced expression of HSP70, a direct indicator of HS, was also observed by HS at 43 and 45 °C in the two cell lines. HS at 45 °C resulted in decreased cell viability as determined using a sensitive CCK-8 cell counting assay. Because the enhanced Tax expression reached a plateau by heating at 43 °C for 30 min, and that HSP70 expression was apparently enhanced at 43 °C, all subsequent studies were carried out with HS treatment at 43 °C.

Next, we determined the optimum exposure time for enhanced Tax expression. As shown in Figure 1b, incubation for 30 min was sufficient for the enhanced expression of both Tax and HSP70 with minimum cytotoxic effect. On the basis of these results, all subsequent studies were carried out using HS at 43 °C for 30 min. It is noteworthy that the MFI for Tax^+^ cells also slightly increased under HS at both bulk and single cell levels as shown in Supplemental Figures S1 and S2.

### 3.2. HS Increases the Total Amount of Tax Protein

**Figure 2:**
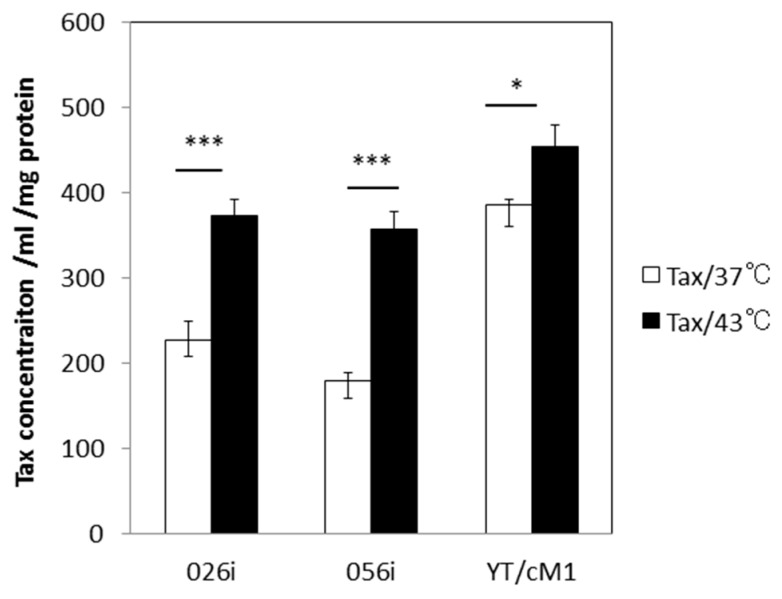
Effect of HS on total Tax protein expression: Aliquots of the ATL-derived cell lines (ATL-026i and ATL-056i) and the HTLV-I-immortalized CD4^+^ T cell line (YT/cM1) were incubated at either 37 °C or 43 °C for 30 min followed by incubation for 24 h in triplicates wells. The cells were then collected, washed, lysed in lysis buffer and the total protein concentrations were determined. Then amounts of total Tax concentration/mg total protein were quantitated using our Tax specific enzyme-linked immunosorbent assay ELISA. The values denote the means ± SD. * *p* < 0.05, *** *p* < 0.001.

The intra-cellular localization of Tax has been shown to be altered in response to various forms of cellular stress, such as HS and ultra violet (UV) light, resulting in an increase in cytoplasmic Tax about 1~2 h after treatment and a decrease in Tax speckled structures [15], which might affect Tax detection by FCM. In order to confirm the enhancing effect of HS on Tax expression, we quantified the levels of total Tax protein in whole cell lysates by using our in-house Tax-specific ELISA. As shown in Figure 2, the levels of Tax protein increased significantly by exposure to HS in three distinct T cell lines including two ATL-derived CD4^+^ T cell lines and an in vitro- HTLV-I-immortalized CD4^+^ T cell line prepared from a normal donor (YT/cM1). These data thus confirm the fact that exposure to HS treatment increases the total amount of Tax antigen per culture during the 24 h culture period.

### 3.3. HS Up-Regulates a Functional Form of Envelope gp46.

**Figure 3:**
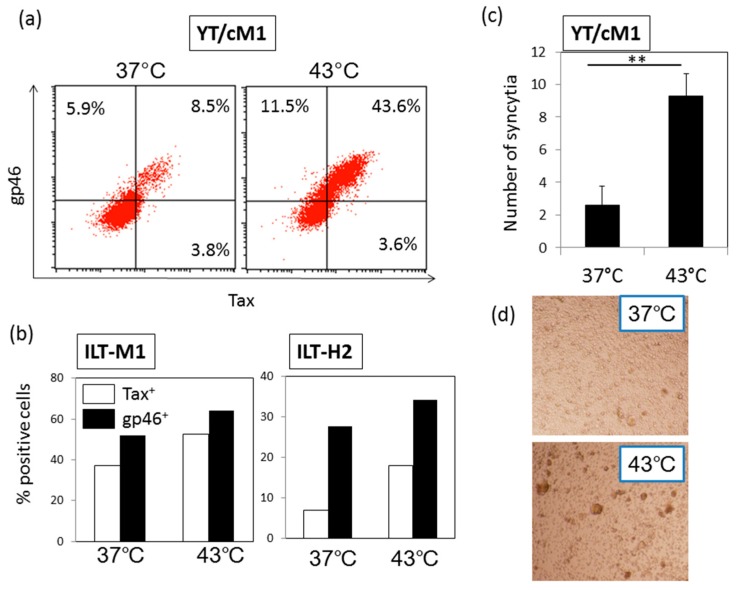
Enhancement of HTLV-I envelope gp46 expression by HS: Three HTLV-I-producing T cell lines CD4^+^ YT/cM1, an HTLV-I associated myelopathy/tropical spastic paraparesis (HAM/TSP)-derived CD8^+^ ILT-M1 and an ATL-derived CD4^+^ ILT-H2, were either exposed to HS or mock treated and cultured for 24 h. (**a**,**b**) The levels of gp46 along with Tax antigen expression were determined by FCM. Data shown are representative of three independent experiments. (**c**) Syncytium formation capacity was compared between untreated and heat shocked YT/cM1 cells by a co-culture method using Jurkat cells as the indicator cell line. The numbers of syncytia were counted in triplicate cultures and the means ± SD are shown. ** *p* < 0.01. (**d**) Syncytia formed in each culture were microscopically observed using an inverted microscope at magnification of 100×.

Next, we examined whether exposure to HS influences the expression of the HTLV-I structural envelope protein gp46 whose expression is known to be enhanced by Tax-mediated transactivation. As shown in Figure 3a, b, the up-regulation of gp46 antigen expression was apparent in three HTLV-I-infected T cell lines including the CD4^+^ and CD8^+^ T cell line (ILT-M1). In Figure 3a, it was obvious that gp46^+^ Tax^−^ cells also increased after HS. We speculate that the gp46 on Tax-negative cells may represent biofilm of HTLV-I particles produced from Tax-positive cells, or alternatively, gp46^+^ Tax^−^ cells may be at a resting phase after a productive infection phase. It is noteworthy that syncytium-forming capacity which is an indicator of HTLV-I infectivity, was enhanced significantly by the exposure of YT/cM1 cells to HS (Figure 3c and Figure 3d), indicating that HS also enhanced the functional form of gp46 expression. In addition, production of gag p24 in the culture supernatant of YT/cM1 cells was also enhanced by HS (Supplemental Figure S3).

### 3.4. Time Course Effects of HS

**Figure 4:**
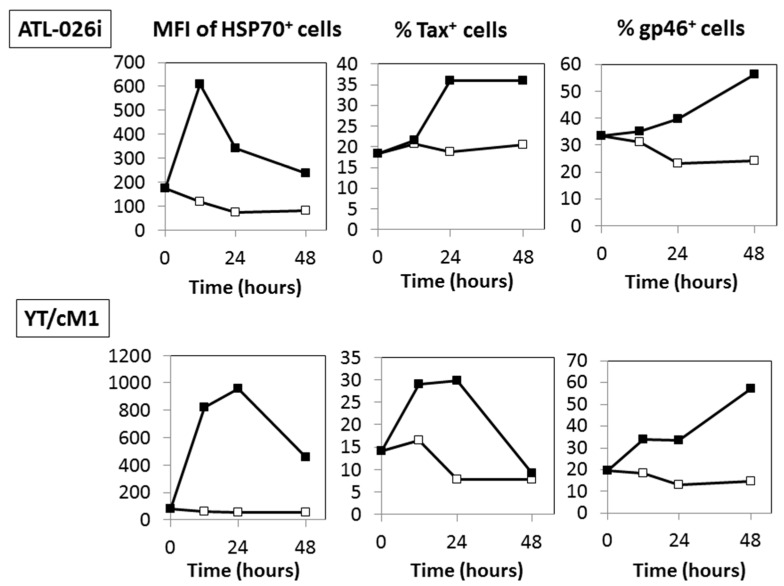
A comparison of time course of the expression among HSP70, Tax and gp46 antigens followed by exposure to HS: The ATL-026i (upper panel) and YT/cM1 (lower panel) cells were either exposed to HS (black square) or mock treated (open square), cultured for 12, 24 and 48 h, and then their phenotypes were analyzed by FCM. Data shown are representative of three independent experiments.

We examined the kinetics by which exposure to HS leads to optimal expression of HSP70, Tax and gp46 using two HTLV-I^+^ T cell lines (ATL-026i and YT/cM1). As shown in Figure 4, there appeared to be a sequential increase in HSP70 expression peaking at during 12~24 h, followed by Tax and then finally gp46 antigen expression. The expression levels of these three antigens returned to control levels by ~72 h (unpublished data, [31]). The delay in enhanced gp46 expression in comparison to Tax antigen suggests that gp46 expression was dependent on Tax antigen expression.

### 3.5. A Possible Involvement of Heat Shock Protein 70 (HSP70)

**Figure 5:**
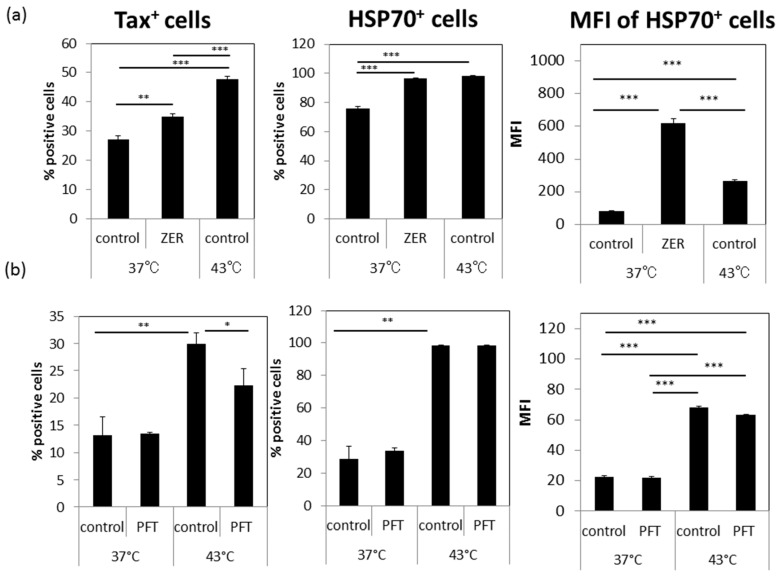
The effects of the HSP70 inducing and inhibiting agents on the expression of Tax antigen: Triplicate cultures of YT/cM1 cells were either exposed to HS or mock treated, and then cultured in the presence or absence of either (**a**) 10 μM zerumbone (ZER, HSP70 inducer) or (**b**) 1 μM pifithlin-μ (PFT, HSP70 functional inhibitor) for 24 h. The Tax and HSP70 expression was analyzed by FCM. The values denote the means ± SD. * *p* < 0.05, ** *p* < 0.01, *** *p* < 0.001.

In attempts to test a possible involvement of HSP70 in the enhanced expression of Tax and gp46, we tested the effects of the HSP70-inducing chemical ZER and the HSP70 functional inhibitor PFT. The results of preliminary studies indicated that the optimal concentrations to be utilized for ZER was noted at 10 μM and PFT at 1 μM without any detectable effect on cell viability and cell growth. As shown in Figure 5a, incubation of cultures with ZER for 24 h significantly up-regulated both the frequencies of Tax^+^ and HSP70^+^ cells in the absence of HS. On the other hand, PFT treatment inhibited the increase in the frequencies of Tax^+^ cells by HS (Figure 5b). HSP70 expression itself was not altered by incubation with PFT since PFT is only a functional inhibitor of HSP70. These results indicate that HSP70 might be involved at least in part in the HS-induced enhancement of Tax antigen expression.

### 3.6. Effect of HS on Tax-Inducible Host Antigens CD83 and OX40

**Figure 6:**
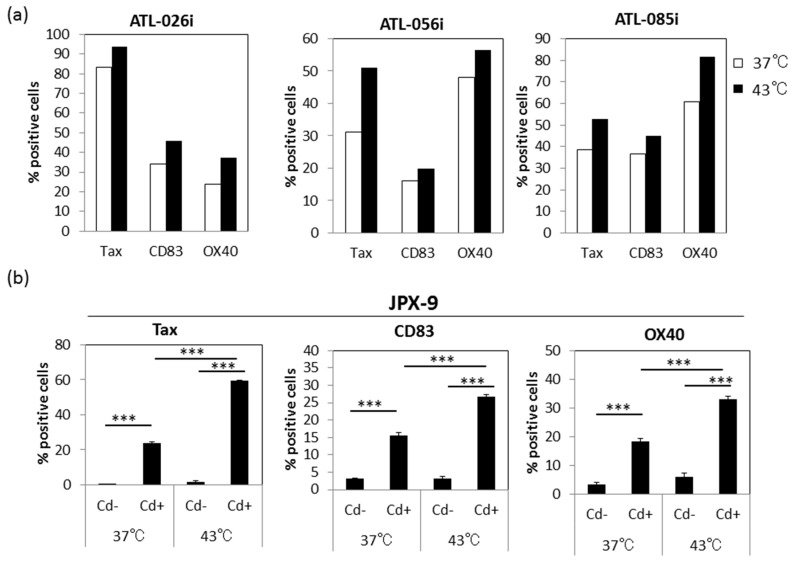
The up-regulation of the expression of Tax-inducible host antigens by exposure to HS: The ATL-derived CD4^+^ T cell lines (**a**) and JPX-9 cell line (**b**) were either exposed to HS or mock treated, and then cultured for 24 h in the presence or absence 1 μM Cd (Tax inducing agent in JPX-9 cells). The frequencies (%) of Tax antigen and Tax-inducible host antigens including CD83 and OX40 expressing cells were analyzed by FCM. Data shown are representative of three independent experiments. In (**b**), experiments were performed in triplicates, with incubation in media alone (Cd-) or media containing Cd (Cd+) and the frequencies of Tax, CD83 and OX40 cells determined. The means ± SD values are shown. *** *p* < 0.001.

Because Tax is known to up-regulate a variety of host antigens, some of which are associated with immortalization or transformation of HTLV-I-infected T cells, we examined the effect of HS on the expression of some of the Tax-inducible host antigens, including CD83 and OX40. As shown in Figure 6a, the levels of CD83 and OX40 expression were significantly up-regulated by exposure to HS in three HTLV-I-infected CD4^+^ T cell lines. Representative dot blots are shown in Supplemental Figure S4. To demonstrate the involvement of Tax in these enhanced CD83 and OX40 antigen expressions, we examined the effect of HS exposure on JPX-9 cells which carry the Cd inducible Tax gene. Figure 6b showed that incubation of the JPX-9 cells in media containing Cd and HS exposure induced not only increased levels of Tax but also CD83 and OX40 on the cell surface. Thus, it was apparent that HS enhanced the expression of not only Tax antigen but also Tax-inducible host antigens.

### 3.7. Effect of HS Exposure on Primary Human CD4^+^ T Cells Infected with HTLV-I

**Figure 7:**
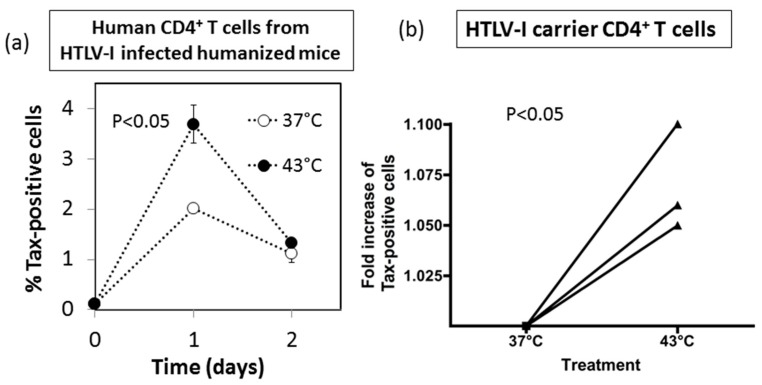
Effect of HS exposure on primary human CD4^+^ T cells infected with HTLV-I: (**a**) NOD/SCID/γc null mice (NOG mice, n=3) were humanized by transplantation with normal human peripheral blood mononuclear cells (PBMCs) and infected with HTLV-I (as described in Materials and Methods). After 2 weeks, cells within the peritoneal lavage were collected and either exposed to HS (black circle) or mock treated (open circle). These cells were cultured in triplicates and examined for the frequencies of Tax antigen expressing cells on day 0, 1 and 2. The values denote the means ± SE. * *p* < 0.05; (**b**) Aliquots of diluted whole blood from three different HTLV-I carriers were either exposed to HS (43 °C) or mock treated, and then cultured for 24 h. The gated population of CD4^+^ T cells was analyzed for the expression of Tax and gp46 antigens by FCM.

Finally, we examined the effect of HS exposure on HTLV-I-infected primary human CD4^+^ T cells, including those obtained from HTLV-I-infected humanized NOG mice and HTLV-I carriers. As shown in Figure 7a, exposure to HS resulted in significant increase in the frequencies of Tax^+^ CD4^+^ T cells after in vitro culture for 24 h (*p* < 0.05), that returned to control levels by 48 h. Figure 7b shows that exposure to HS resulted in increase in the expression of Tax antigen in primary CD4^+^ T cells from three HTLV-I carriers (*p* < 0.05). Representative dot blots are shown in Supplemental Figure S5. Taken together, these data support the view that HTLV-I activation by exposure to HS is a general phenomenon rather than restricted to having effects on long-term cultured cell lines.

## 4. Discussion

In the present study, we demonstrated that exposure to HS up-regulates the expression of HTLV-I Tax antigen during in vitro culture for 12~48 h along with induction of functional HTLV-I gp46 and the Tax-inducible host antigens CD83 and OX40 in not only human HTLV-I-infected T cell lines but also in primary human CD4^+^ T cells. We conclude from these observations that HS is one of the environmental factors that can potentially activate integrated HTLV-I provirus in vitro in latently infected T cells, although in vivo studies are yet to be performed.

In the literature, Andrew et al. reported that heat stress enhances the expression of HTLV-I envelope and gag antigens in long-term cultured HTLV-I-transformed T cells lines, such as HUT-102 and MT-2 cells [14,32,33], and suggested that the enhancement is due to increased translation of HTLV-I structural proteins, but not changes in protein turnover. Since the synthesis of viral structural proteins of HTLV-I is dependent on its Tax protein, our present study suggested that Tax antigen synthesis precedes that of HTLV-I structural proteins. Indeed, results of the kinetic studies showed that the enhanced gp46 antigen expression came after enhanced Tax antigen expression (Figure 4). As expected, enhanced Tax antigen expression was accompanied by enhanced expression of some of Tax-inducible host antigens (Figure 6), indicating that enhanced Tax antigen molecules are functional. In addition, in line with the fact that HS is able to enhance HIV-1 Tat-mediated transactivation of the LTR independently of Tat expression levels, there might be an alternative mechanism for Tax-mediated enhancement of HTLV-I production upon HS.

The precise mechanism by which Tax antigen synthesis is up-regulated by exposure to HS is yet to be defined. On the basis of our preliminary data (data not shown) indicating that HS up-regulated total levels of Tax mRNA, it is possible that HS directly controls transcription. Thus, further studies are in progress to determine this possibility by measuring nuclear RNA levels for Tax RNA and genomic RNA in the cytoplasm. Since Tax operates by the activation of transcriptional factors such as the cAMP-response element-binding protein (CREB) for activation of not only viral but also host cellular genes, it can be speculated that CREB is also activated by exposure to HS in HTLV-I-infected cells. The finding that ER stress is induced by exposure to HS [34] and in turn CREB is activated by ER stress in HeLa cells supports this view [35]. However, our preliminary FCM experiments showed that HS did not affect the levels of expression of phosphorylated forms of NF-kB and CREB molecules (Supplemental Figure S6).

As it is the case that the cellular onco-protein c-Myc protein is up-regulated following HS [36], rapid induction of Tax antigen after heat-stress may be an advantage of HTLV-I-infected cells for induction of the recovery process. In addition, enhanced Tax expression resulting in enhanced infectious HTLV-I viral production may also be an advantage for the spread of HTLV-I from heat-stressed cells. It is likely that HTLV-I infected cells after HS may become good targets for HTLV-I Tax-specific CTL [37,38] or ADCC by anti-gp46 antibodies plus NK cells [26]. However, CTL and NK cell activities are suppressed when exposed to temperatures >41 °C and 42 °C, respectively, [39,40]. We have confirmed that exposure to HS inactivates ADCC activity of PBMCs against HTLV-I-infected cells (unpublished data, [41]). Therefore, heat-stress may favor HTLV-I infection rather than immune-surveillance against HTLV-I at a local site of exposure to HS. Further studies to reveal molecular mechanisms underlying this HS-mediated HTLV-I activation are yet to be performed.

Another representative cellular stress that is critical for Tax expression is oxidative stress, such as As_2_O_3_. It has been utilized for ATL therapy as it induces the apoptosis of HTLV-I-infected cells [42]. The specificity of the effect of As_2_O_3_ on HTLV-I synthesis has been a subject of controversy. Thus, while Andrew et al. reported an enhancing effect [14,32,33], Nabeshi et al. in contrast showed a suppressing effect [43]. These discrepancies seem to be due to different experimental conditions. In our preliminary studies, we could not observe As_2_O_3_ mediated enhancement of Tax and gp46 expression in various conditions and various types of cell lines (Supplemental Figure S7). Thus, it is likely that HS stress and As_2_O_3_-mediated oxidative stress may utilize distinct pathways on Tax expression by HTLV-I infected cells.

The involvement of HSPs in viral infection has been reported for several viruses [44,45,46] including HTLV-I [19,47,48,49,50]. Our present data indicates that among the HSP family, HSP70 might be associated at least in part with HS-mediated enhancement of Tax antigen expression based on the data obtained with the use of the HSP70-inducer, ZER, and the HSP70 functional inhibitor PFT (Figure 5). The increased numbers of HSP70 molecules after exposure to HS may serve to function as molecular chaperon for Tax and the other HTLV-I antigen molecules together with the other constitutively expressed HSPs including HSP90 [49]. Further studies are in progress to determine what types of HSP are involved in the HS-mediated enhanced Tax expression.

Infrared sunlight is known to generate heat and for their ability to increase skin temperature depending on exposure dose and time. It has been reported that intra-dermal-temperature reaches up to 44 °C when irradiated with IR at 970 nm at 80 mW/cm^2^ within 15 min [51]. Thus, it can be speculated that exposure to direct sunlight in subtropical or tropical areas might raise the skin temperature to above 43 °C resulting in HS of HTLV-I-infected cells. In addition, skin inflammation generated by exposure to strong solar UV radiation (sunburn) may cause the recruitment of HTLV-I-infected cells to the sites of inflammation. Furthermore, systemic physiological hyperthermia induced by either direct exposure to sunlight or an opportunistic infection (for example, infection with Strongyloides stercoralis) might also stimulate HTLV-I expression in vivo, as is the case for HIV-1 [52]. Although we failed to demonstrate that activation of HTLV-I-infected CD4^+^ T cell lines via CD3 molecule further enhanced the effect of HS on Tax expression (Supplemental Figure S8), it is also possible that immune activation of fresh HTLV-I-infected T cells in vivo might synergize HS.

In this way, it is likely that, in subtropical and tropical areas, repeated skin exposure to strong sunlight for a prolonged time can cause HTLV-I reactivation in vivo.

## 5. Conclusions

In conclusion, results of the present study indicate that a mild exposure to heat shock may be one of the natural environmental factors that stimulate Tax antigen expression leading to not only reactivation of HTLV-I in latently infected T cells but also induction of expression of Tax-inducible host antigens, which may favor expansion of HTLV-I infection in vivo. It can be speculated that exposure to strong sunlight in subtropical and tropical areas is involved in the geographic bias of increased HTLV-I prevalence within these regions, and that, for HTLV-I carriers, precaution against exposure to strong sunlight for prolonged periods of time might be beneficial to prevent reactivation of HTLV-I.

## Supplementary Materials

The following figures are available online at www.mdpi.com/1999-4915/8/7/191/s1. **Figure S1.** HS increased the MFI of Tax^+^ cells. **Figure S2.** Correlation of Tax and HSP70 expression; **Figure S3.** HS increased p24 concentration; **Figure S4.** Correlation between intra-cellular Tax and cell surface expression of OX40 and CD83; **Figure S5.** HS upregulated expression of Tax and gp46 in fresh PBMC from careers; **Figure S6.** Effect of HS on the expression of NF-kB and CREB molecules; **Figure S7.** The failure of exposure to oxidative stress, As_2_O_3_, to induce increased expression of Tax and gp46; **Figure S8.** Failure to stimulate HTLV-I-infected cell lines with anti-CD3 mAb (OKT-3).
