# Supplementary material for: Heat Shock Enhances the Expression of the Human T Cell Leukemia Virus Type-I (HTLV-I) Trans-Activator (Tax) Antigen in Human HTLV-I Infected Primary and Cultured T Cells
Source: Viruses. 2016 Jul 9;8(7):191. doi: 10.3390/v8070191 (PMC4974526; doi:10.3390/v8070191)
Supplement: Supplementary file 1 [file viruses-08-00191-s001.pdf]

# Supplementary Materials: Heat Shock Enhances the Expression of the Human T Cell Leukemia Virus Type-I (HTLV-I) Trans-Activator (Tax) Antigen in Human HTLV-I-Infected Primary and Cultured T Cells

**Marie Kunihiro, Hideki Fujii, Takuya Miyagi, Yoshiaki Takahashi, Reiko Tanaka, Takuya Fukushima, Aftab A Ansari, and Yuetsu Tanaka**

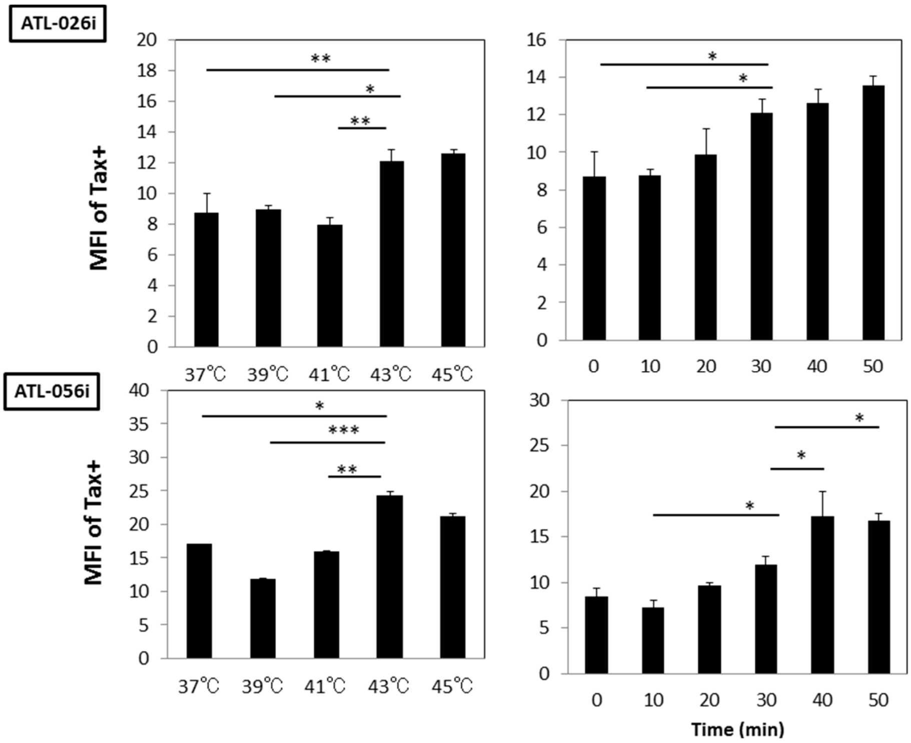


**Figure S1.** HS increased the MFI of Tax⁺ cells: HTLV-I-infected cell lines, ATL-026i and ATL-056i, were either mock treated (37 °C) or exposed to heat stress at 39-45 °C or for 30 min or at 43 °C for 10-50 min. Tax expression at 24 h after heat shock (HS) is shown. The values denote the means ± SD. *$p<0.05$, **$p<0.01$, ***$p<0.001$.

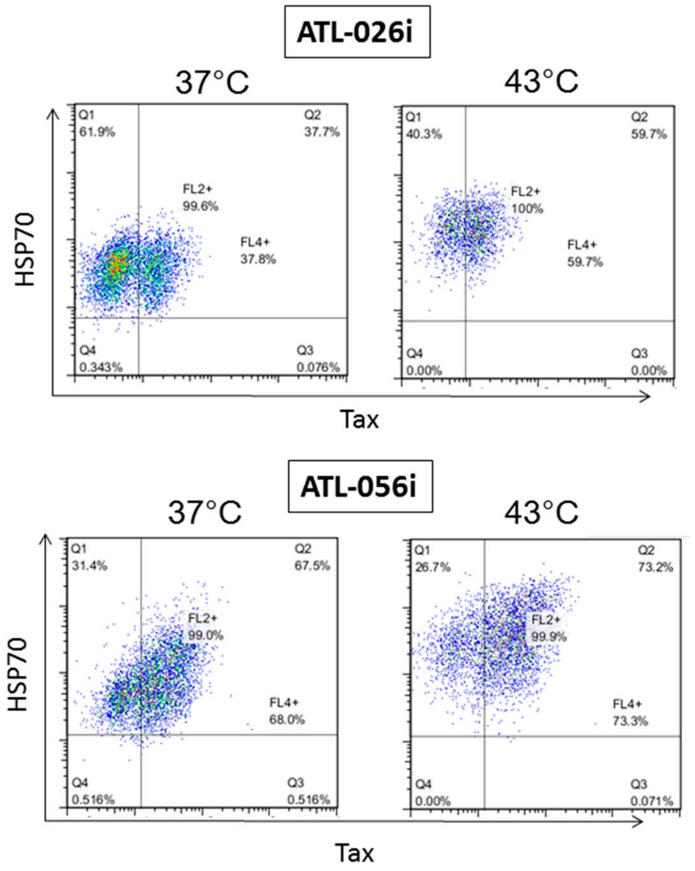


**Figure S2.** Correlation of Tax and HSP70 expression: The HTLV-I-infected cell lines, ATL-026i and ATL-056i, were either mock treated (37 °C) or exposed to heat stress at 43 °C for 30 min. Tax and HSP70 expression one day after HS are shown in dot plot.

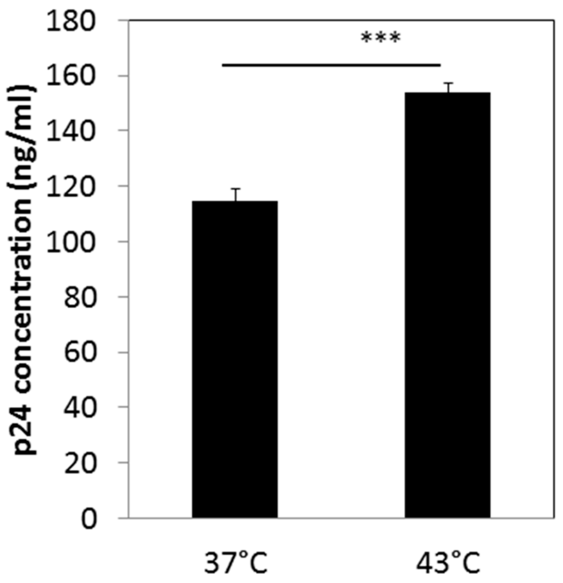


**Figure S3.** HS increased p24 concentration: The HTLV-I-infected cell lines, YT/cM1 was either mock treated or heat shocked. Concentration of p24 in culture supernatant was measured by ELISA at two days after HS. The values denote the means ± SD. ***$p<0.001$.

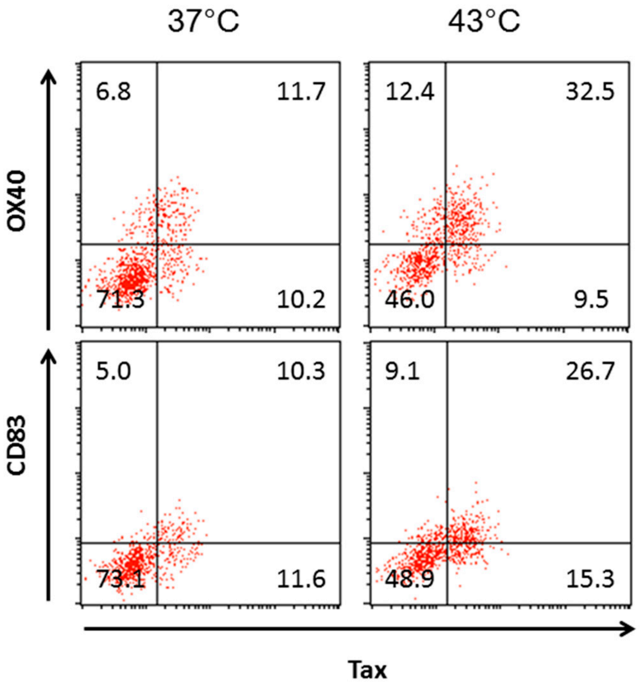


**Figure S4.** Correlation between intra-cellular Tax and cell surface expression of OX40 and CD83: The HTLV-I-infected cell line, ATL-026i, was mock treated (37 °C) or heat shocked at 43 °C or for 30 min. After 24 h cultivation, cells were analyzed for simultaneous expression of Tax, OX40 and CD83.

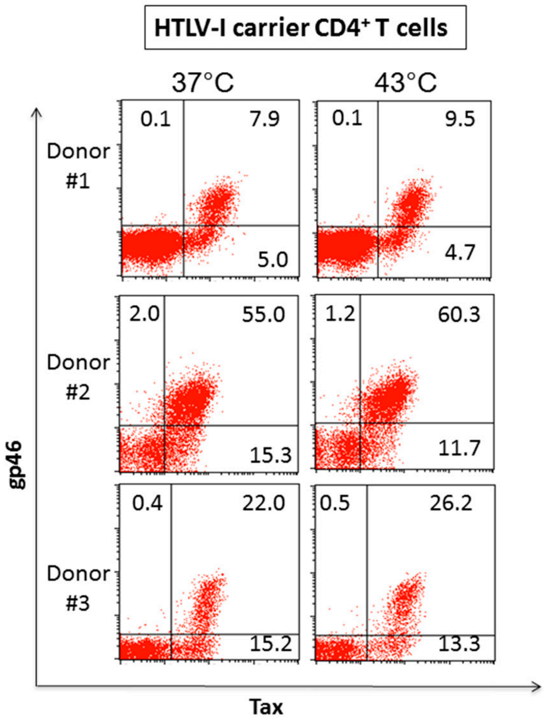


**Figure S5.** HS upregulated expression of Tax and gp46 in fresh PBMC from careers: Aliquots of diluted whole blood from three different HTLV-I carriers were either exposed to heat shock (43 °C) or mock treated, and then cultured for 24 h. The gated population of $CD4^{+}$ T cells was analyzed for the expression of Tax and gp46 antigens by FCM.

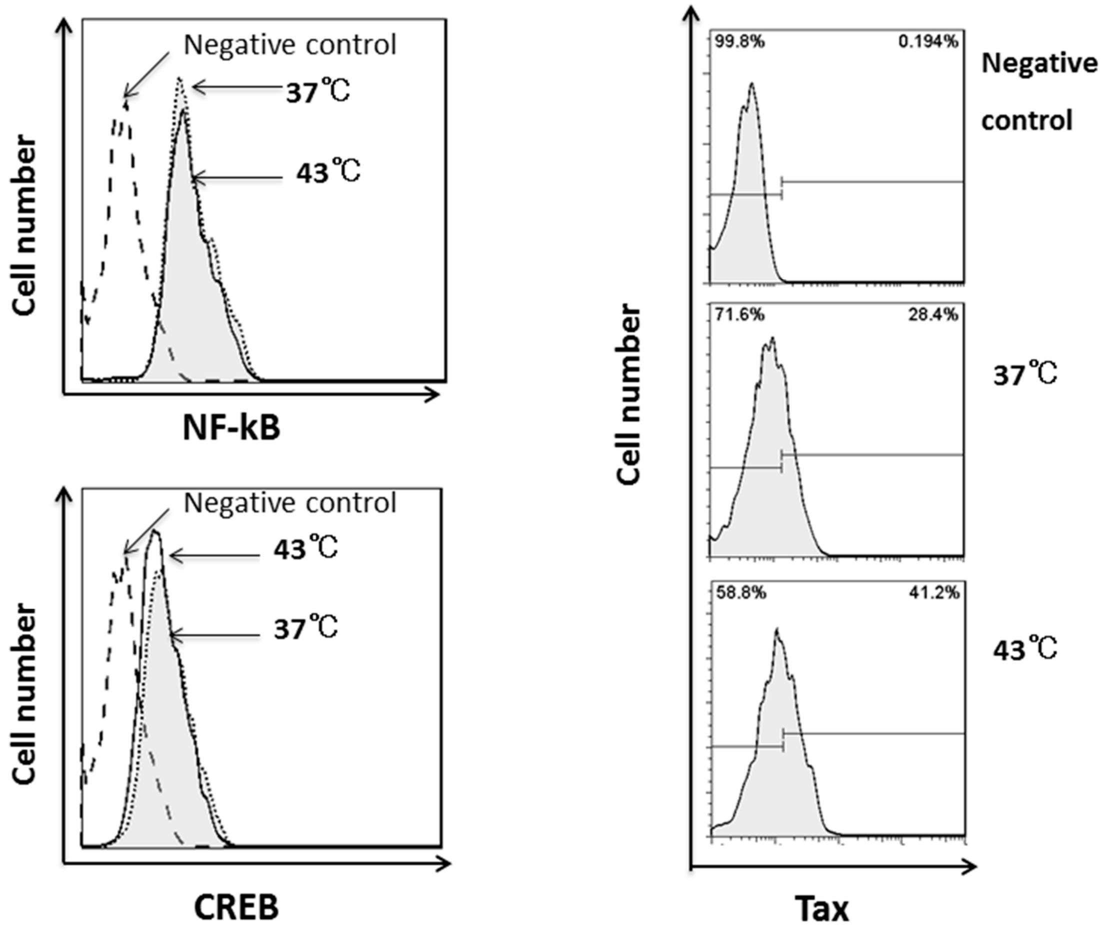


**Figure S6.** Effect of HS on the expression of NF-kB and CREB molecules: HTLV-I-infected cell line ATL-056i was mock treated (37 °C) or heat shocked. After 24 h incubation, the intracellular expression of NF-kB and CREB was analyzed by FCM (left panel). Primary antibodies used were rabbit mAbs anti-NF-kB p65 antibody (E379) and anti-CREB antibody (E306) purchased from Abcam (Cambridge, MA, USA) followed by staining with donkey anti-rabbit IgG-FITC (Biolegend, SanDiego, CA, USA). Negative control showed staining with only 2nd anti-rabbit IgG antibody. Tax expression of these cells is shown in right panel.

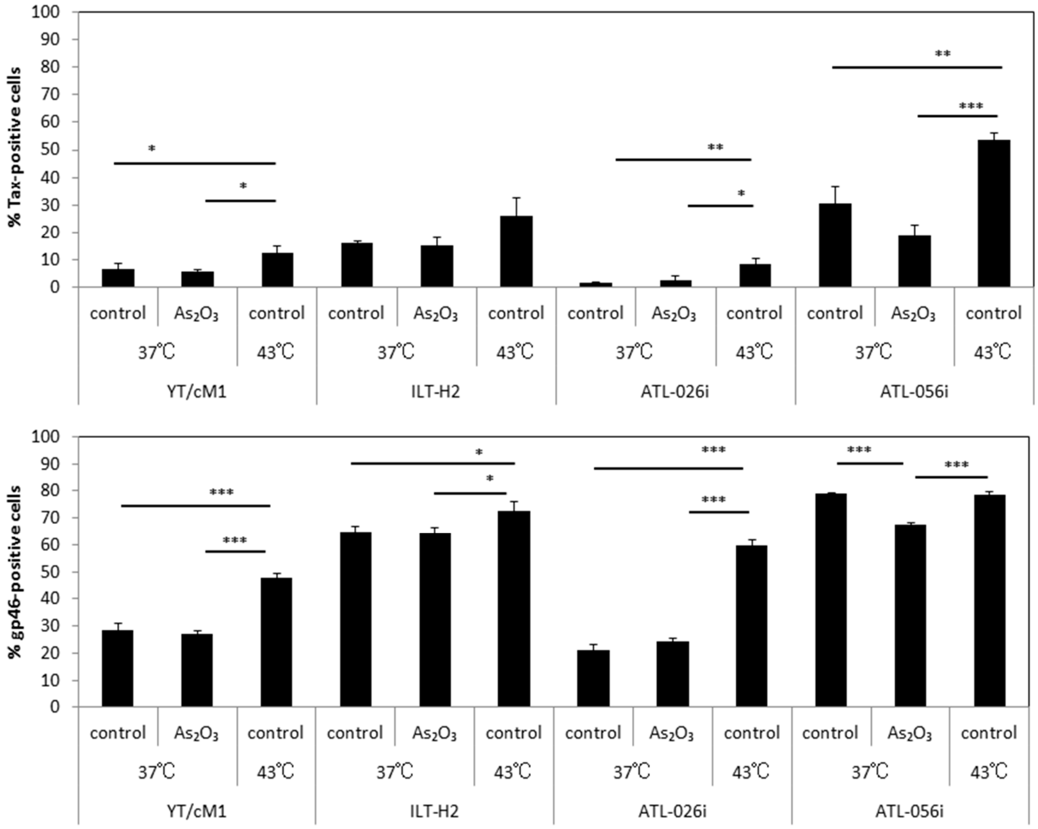


**Figure S7.** The failure of exposure to oxidative stress, $As_2O_3$, to induce increased expression of Tax and gp46: The HTLV-I-infected cell lines YT/cM1, ILT-H2, ATL-026i and ATL-056i, were either mock treated or exposed to heat stress at 43 °C for 30 min or oxidative stress in the presence of 0.25~1.0 μM $As_2O_3$, and then cultured for 24 h. The frequencies of Tax and gp46 expression were analyzed by FCM. The values denote the means ± SD. *$p$<0.05, **$p$<0.01, ***$p$<0.001.

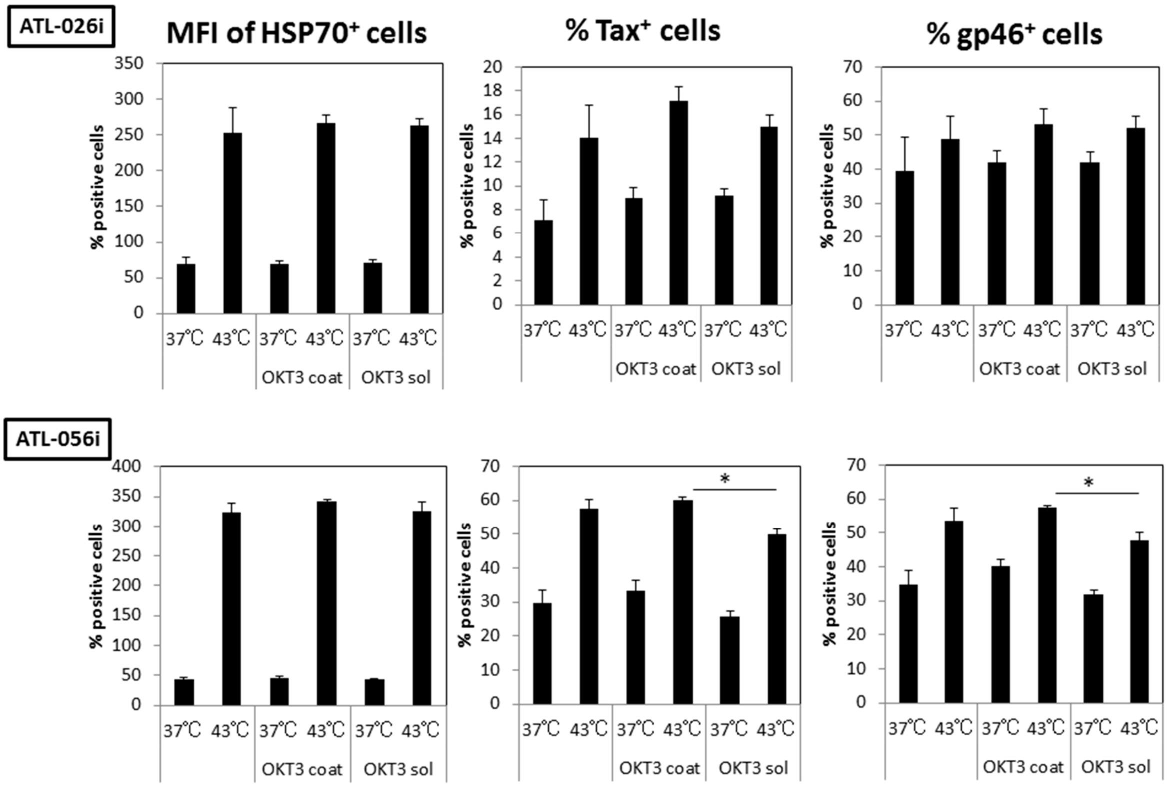


**Figure S8.** Failure to stimulate HTLV-I-infected cell lines with anti-CD3 mAb (OKT-3): ATL patient-derived HTLV-I-infected cell lines, ATL-026i and ATL-056i, were mock treated (37 °C) or exposed to heat stress, cultured alone or in the presence of either immobilized OKT-3 (OKT-3 coat) or soluble OKT-3 (2 μg/mL) (OKT-3 coat) for 24 h. Then, Tax, gp46 and HSP70 expression were analyzed by FCM.

© 2016 by the authors, licensee MDPI, Basel, Swizerland. This article is an open access article distributed under the terms and conditions of the Creative Commons Attribution (CC-BY) license (http://creativecommons.org/licenses/by/4.0/).
